# Discovery of a Biological Mechanism of Active Transport through the Tympanic Membrane to the Middle Ear

**DOI:** 10.1038/srep22663

**Published:** 2016-03-07

**Authors:** Arwa Kurabi, Kwang K. Pak, Marlen Bernhardt, Andrew Baird, Allen F. Ryan

**Affiliations:** 1 University of California San Diego, Department of Surgery/Otolaryngology, La Jolla, CA, 92037, USA; 2 University of California San Diego, Department of Surgery/Trauma, La Jolla, CA, 92037, USA; 3 Universitätsklinik Würzburg, Department of ENT, Würzburg, 97070, Germany; 4 Veterans Affairs San Diego Healthcare System, San Diego, CA, 92161, USA

## Abstract

Otitis media (OM) is a common pediatric disease for which systemic antibiotics are often prescribed. While local treatment would avoid the systemic treatment side-effects, the tympanic membrane (TM) represents an impenetrable barrier unless surgically breached. We hypothesized that the TM might harbor innate biological mechanisms that could mediate trans-TM transport. We used two M13-bacteriophage display biopanning strategies to search for mediators of trans-TM transport. First, aliquots of linear phage library displaying 10^10th^ 12mer peptides were applied on the TM of rats with active bacterial OM. The middle ear (ME) contents were then harvested, amplified and the preparation re-applied for additional rounds. Second, the same naïve library was sequentially screened for phage exhibiting TM binding, internalization and then transit. Results revealed a novel set of peptides that transit across the TM to the ME in a time and temperature dependent manner. The peptides with highest transport capacities shared sequence similarities. Historically, the TM was viewed as an impermeable barrier. However, our studies reveal that it is possible to translocate peptide-linked small particles across the TM. This is the first comprehensive biopanning for the isolation of TM transiting peptidic ligands. The identified mechanism offers a new drug delivery platform into the ME.

Otitis media (OM) is a major health problem in the US and worldwide being the most frequently reported pediatric infection with up to 85% of children experiencing at least one episode before the age of three^1^,^2^. Currently, OM is the most frequent cause of antibiotic prescriptions and surgery in children^3^,^4^. In addition to the pain and distress of middle ear (ME) infection, chronic or recurrent OM can cause conductive or even sensorineural hearing loss. Hearing loss due to OM has been linked to deficits in speech perception, language delays and learning disabilities^5^. Moreover, in developing countries where access to health care is limited, the burden of mortality or hearing loss due to OM is greater: in 2004, the WHO estimated that undertreated OM causes 28,000 annual deaths, and half of the world’s burden of severe hearing loss^5^.

*Streptococcus pneumoniae, Haemophilus influenzae*, and *Moraxella catarrhalis* are the three most common bacteria isolated from the MEs of children with OM^6^. The typical treatment is systemic antibiotics^1^, but there are problems associated with this therapy. Side-effects include gastrointestinal distress and diarrhea. More seriously, a rise in antibiotic-resistant bacteria has been attributed to the overuse of antibiotics^7^,^8^. Moreover, Pichichero *et al*.^9^ reported that 10–15% of patients receiving oral amoxicillin showed no detectable drug levels in the ME. While the use of antibiotics is no longer recommended as standard treatment for acute, uncomplicated OM, they remain a mainstay of therapy for chronic/recurrent OM^10^. Streptococcal vaccines have been developed to reduce OM due to covered strains, but those have increased incidence for other microbes^11^,^12^. Surgical treatments like myringotomy and tympanostomy tubes are helpful in persistent disease intervention but there is controversy about long-term benefit and later TM abnormalities^13^,^14^. In addition, these procedures require surgical training and expertise, involve general anaesthesia in children, and are expensive. As noted above, in many underprivileged parts of the world the access to surgical and even basic otologic care can be lacking, leading to severe complications like hearing loss and even death from undertreated infections^5^. Hence, there is great need to develop novel therapeutic agents for OM treatment. Local drug treatment could be effective, and help overcome many of the hurdles discussed above. However, local therapy is limited by the impermeable nature of the TM that separates the external ear canal from the ME.

The primary function of the TM is to collect and transmit sound from the ear canal through the ossicles of the air-filled ME to the inner ear. It also functions as a barrier to air pressure and fluids, and protects the ME from foreign substances and infective organisms^15^,^16^. The human TM consists of three layers: an outer layer of keratinized epithelium that is continuous with that of the external canal skin; a middle layer of fibrous connective tissue consisting of radial and circumferential fibers that provide the majority of the mechanical characteristics and thickness of the membrane, and an inner epithelial layer continuous with that of the ME mucosa^17^,^18^. Its thickness varies from 100–150 μm, depending on location. The majority of the TM consists of the pars-tensa, where the manubrium of the malleus is inserted and which transmits acoustic pressure variations from the external ear canal to the auditory ossicles. The smaller and less stiff pars-flaccida (3–6% of the total area) aids in regulating pressure within the ME^19^. During OM, fluid (an effusion) accumulates behind the TM and the inner mucosa exhibits hyperplasia in some areas. Although infection may leave the TM normal in structure, OM is also associated with a bulging TM and buildup of purulent material due to ME inflammation^20^.

The impermeable nature of the TM is determined largely by its epithelia, since the connective tissue fibers of the middle layer do not appear to form a continuous layer at the molecular level, which isolate the ME from the external ear canal^17^. However, the cells of both the outer and inner epithelia of the TM are connected to one another by tight junctions^17^,^21^. These junctions maintain the TM’s air, fluid and molecular barrier function. As a consequence, the TM is impenetrable to most substances, including the drugs that are commonly locally used to treat diseases of the ear. Therefore, local application of drugs to the middle and inner ear requires disruption of TM integrity, either by needle injection or by insertion of a tympanostomy tube that provides a relatively stable opening as recently demonstrated by Koulich *et al*.^22^. These procedures require time and skillful expertise. Thus, it would be useful to overcome these limitations for simple and local topical therapeutic applications.

Phage display was originally developed to study protein-ligand interactions^23^ but this technology has been utilized in various applications from novel ligand identification^24^, epitope mapping to antibody engineering^25^, and cell/tissue targeting^26^,^27^,^28^. Phage display represents a potentially promising approach for identifying targets for the transport of molecules and particles since the identified ligands are target-specific in confirmation. Phage display typically involves the insertion of random oligonucleotides into a phage genome such that they direct a bacterial host to express large libraries of peptides linked to phage coat proteins (e.g., filamentous phage pIII, pVI or pVIII). The mature phage coat protein incorporates the short fusion peptides resulting in display of an individual exogenous peptide sequence on the exterior surface of each phage particle^29^. Large libraries expressing peptides can be screened through multiple rounds for target affinity and specificity. Phage display has been applied to identify peptide targets that cross the blood brain barrier^30^, the lung endothelium^31^, and the intestinal epithelium^32^.

In this study, we hypothesized that proteins might mediate a biological mechanism of active transport across the TM, and that sequential selection of phage-display libraries would identify rare but novel peptides that actively cross into the ME without damaging the TM. The concept was that phage transported to the ME side, opposite the TM, would display a combination of a transporter peptide linked to an active agent cargo (the phage). Our goal was to identify new and novel targeting and transporter molecules that suit both the challenging drug delivery characteristics and the natural physiology of the ear.

## Materials and Methods

### Phage library and bacteria

The Ph.D-12^TM^ Phage Display Peptide Library (New England Biolabs, Cambridge, MA, USA) was used to perform *in vivo* phage display in our rat model of bacterial OM. In this library the pIII protein displays linear 12mer peptides with a diversity of 2.9 × 10^9^ unique peptide sequences, at about 100 copies per sequence, in 10 μL of supplied phage. Tetracycline resistant *E. coli* ER2738 host bacteria were used for filamentous phage infection to allow for the blue/white screening using IPTG/X-gal plates for all the titration steps. The M13KE wild-type (WT) “empty” phage was used as a control for the determination of unspecific TM binding, ME internalization and phage transport. The WT phage is the M13KE phage in which the *pIII* gene does not code for a random linear 12mer peptide sequence.

### Animal model of OM

All animal experiments were performed in strict accordance to the Guide for the Care and Use of Laboratory Animals of the National Institutes of Health (NIH) and the policies of the Veterans Affairs San Diego Healthcare Systems, CA. Experiments were carried out in strict accordance with an approved Institutional Animal Care and Use Committee (IACUC) protocol. Male Sprague–Dawley rats were purchased from The Jackson Laboratory. The animals were anesthetized with an intraperitoneal injection of 1.0–1.5 ml per 200–300 g bodyweight of rodent cocktail (40 mg/Kg Ketamine, 10 mg/Kg Xylasine, and 0.75 mg/Kg Acepromazine). The skin of the neck was shaved and treated with Povidone antiseptic solution. OM was induced by infecting the ME with nontypeable *Haemophilus influenzae* (NTHi) in a laboratory setting by surgically exposing the bulla via a ventral approach through a vertical midline incision made in the neck. A 25-gauge needle was used to fenestrate the bulla and inject ~50 μL saline solution containing 10^4^ CFU/mL NTHi bacteria strain 3655 (biotype II, originally isolated from the ME of a pediatric OM patient). Following the inoculation, the opening was closed and incision was stapled. The TMs were visually confirmed to be intact after surgery and the rats were given Lactated Ringer’s solution and buprenorphine subcutaneously postoperatively. For *in vivo* phage display experiments, rats were used at two days post NTHi inoculation. The ears were examined otoscopically at this time to confirm signs of active infection and the presence of fluid in the ME cavity.

### Identification and analysis of phage that can cross the TM into the ME

#### Phage identification strategy 1: Direct sequential enrichment for TM transit ability

In the first phage selection strategy, 50 μl aliquots of the naïve phage library, each containing 1,000 copies of the 2.9 × 10^9^ different peptide sequences in the library, were placed on the outer surface of the TM of anesthetized and immobilized rats with active OM, then laid on their side for 1 hr. The remaining phage solution was removed by wicking and the TMs were then extensively washed with PBS several times to remove any unbound phage particles from the external canal and reduce the possibility of ME sample contamination. The animals were then sacrificed by decapitation under deep anesthesia to isolate the ME from the single side where phage was applied. The bullas of the rats were then dissected, isolated, and further rinsed in PBS prior to opening, as another precaution against contamination. The bulla then was opened and ME effusion was collected in a sterile tube for further analysis. The number of phage particles present in the ME fluid sample was assessed using an aliquot of supernatant in standard plaque assay^29^. Phage in the remaining fluid were amplified by infecting bacteria via standard methods^29^ to generate a library of candidate TM-translocating phage, which was re-applied onto the TMs again using the protocol described above. The screen was successively repeated twice before DNA sequencing of 22 randomly selected colonies revealed the collapse of displayed peptides to consensus sequences. Two animals were used per cycle and the samples were pooled before amplification.

#### Phage identification strategy 2: Sequential enrichment of phage for binding to the TM, internalization into the TM and then TM transit

The second *in vivo* screening model used consecutively increasing stringencies of binding, internalization and translocation in each round of selection. In the first stage, the commercial phage library was applied onto the TM of ears with OM, allowed to incubate for 1 hour (hr), and the external canal was extensively rinsed with PBS to remove unbound phage as described earlier. The TM was then harvested from the ME bulla, washed with PBS, and homogenized. Phage present in the homogenate was amplified, and the process was repeated twice to generate a library enriched for phage with TM-binding activity. We then exposed TMs of infected rats to this preselected library, followed by elution with low pH buffer to remove all phage that had not internalized into the membrane. The TM was again harvested by dissection, homogenized, and phage libraries were amplified in 3 consecutive rounds. The resulting “internalizing phage” selected library was again applied to the TM surface of ears for 1 hr in rats with OM. Fluids were then recovered from the opposite ME cavity side, phage within the samples were amplified, and re-applied for three additional rounds. 30 phage particles from the final two rounds of *in vivo* selection were chosen at random and sequenced to identify the peptides displayed by the transported phage. Using this successive selection procedure (ten rounds total), additional populations of peptide-targeted phage with the ability to bind, internalize and finally transit the TM were identified. Two animals per condition were used. Pooled samples recovered from the final round of *in vivo* selection were sequenced.

### Validation of internalization, and comparative peptide phage transport

After three or ten rounds of subtraction/selection in strategies 1 and 2 respectively, single blue plaques were selected at random for preparation of purified stocks and sequencing as recommended by the manufacturer (New England Biolabs, Cambridge, MA, USA). Phage transit efficiency was determined by incubating 1 × 10^10^ PFUs (plaque-forming units) of specific phage over the TMs of rats *in vivo* for 1 hr. At least six animals per time point were used. Incubation, washings, and elution conditions were performed as above. The phage transiting efficiency was determined by comparing the number of plaque-forming phage recovered from the ME fluid (output) by the number of plaque-forming phage in the input (TM side). Purified M13 WT phage, which does not display a peptide, was used for negative controls.

### Transport kinetics, saturation and competitive binding analysis

The phage peptide with one of the highest transport capacities measured above TMT-3 SADSTKTTHLTL, was selected for further transport specificity, kinetics and competitive binding assays. To evaluate the kinetics of transport of the TMT-3 single phage clone, a TMT-3 phage preparation was incubated on the TM over a 1, 2 or 4 hr time period at 1 × 10^10^ PFU concentration. At least six animals per time point were used. Incubation, washing, and elution conditions were performed as above in rats with OM. The level of phage transport was evaluated by determining the titer (PFUs) of TMT-3 phage present in the ME fluid. To assess transport saturation, the recovery of TMT-3 phage from the ME was compared following application of 10^10^ versus 10^12^ phage particles to the TM for 1 hr. The corresponding free peptide SHSADSTKTTHLTLGGG was synthesized by Genscript (Piscataway, NJ, USA) in addition to an untargeted randomized control peptide SHDLSTSATLTHTKGGG (Genscript, Piscataway, NJ, USA) at purity >95%. All peptides were reconstituted in PBS pH, 7.2. For the competition assay TMT-3 phage application to the TM was performed at titer of 10^10^ PFUs, as above. However, 1 μM of free peptide was included with the phage. Assuming five molecules of pIII-displayed peptide on each virion^29^, the free peptide was used at a molecular excess of 1 μM.

### DNA sequencing, peptide sequence analysis

DNA sequencing to determine the peptide insert sequences encoded on the pIII phage protein was performed as recommended by the manufacturer (New England Biolabs, Cambridge, MA, USA). After the *in vivo* biopanning, 20 to 30 phage clones were randomly selected from titered phage plaques. An over-night culture of *E. coli* ER2738 was diluted 1:100 in LB medium and then 1 mL aliquots were generated and inoculated with each clonal phage. The inoculates were cultured at 37 °C for 5 hrs with shaking. The single-stranded phage DNA (ssDNA) was prepared with a commercial kit (Spin M13 Kit; Qiagen, Valencia, CA, USA). After quantification of yields on agarose gels, DNA sequencing was performed using primers M13-96 gIII supplied by the library manufacturer and the sequencing service of Retrogen Inc. (San Diego, CA, USA). Online bioinformatics server ExPASY was used to convert the DNA sequences to amino acids.

### Data analysis

Peptide sequence data was aligned and dendrograms constructed using MacVector protein sequence analysis software (Eastman Chemical Co., New Haven, CT, USA). Homology analysis was carried out using BLASTP (NCBI) and sequence alignments using ClustalW2 (http://www.ebi.ac.uk/Tools/ msa/clustalw2). The ExPASY Bioinformatics Resource Portal (Swiss Institute of Bioinformatics) was used for predicting peptide properties and parameters. PepDraw (Tulane University) was used to generate peptide primary structures. Web log (http://weblogo.berkeley.edu/) was used to generate the consensus Logo plots showing conserved amino acids at specific positions. The deduced amino acid sequences were applied into the SAROTUP suite and MimoDP web tool to identify any target-unrelated peptides or if these peptides have been identified in previous biopanning screens by other research groups^33^,^34^.

Differences in the mean *A* value, number of eluted phage particles, were compared using a one-way analysis of variance (ANOVA) or two-sided Student’s *t*-test with unequal variance. Statistical significance was assessed at the level of *P* = 0.01. All results were presented as mean ± SD of mean unless otherwise noted. In most experimental cases, the group size was six rats unless indicated.

## Results

### Identification and analysis of phage that can cross the TM into the ME

Prior to proceeding with the *in vivo* phage display TM screening, it was important to develop an animal model system to study our hypothesis. As our goal was to identify peptides that permit or facilitate the transport of phage across the TM for treatment of OM, we used an OM animal model (in rats) to perform the phage screen *in vivo* with un-perforated TM. Using an infected ME experimental model allowed us to withdraw the ME effusions/fluids after isolating and rinsing the bulla, and to analyze for phage transport from the external tympanic cavity into the ME quantitatively after a preset time. Aliquots of the ME effusions were tittered followed by plating and quantitating phage plaques. In addition, analysis of lavage fluid from a contralateral infected ear showed no presence of phage particles by our standard plaque assay, indicating that the phage transport observed was a local rather than a systemic outcome. Untargeted WT phage was always used as an experimental negative control to exclude the possibilities of contamination, TM microperforations or nonspecific transport.

#### Phage identification screening strategy 1: Direct sequential enrichment for TM transit ability

**Figure 1:**
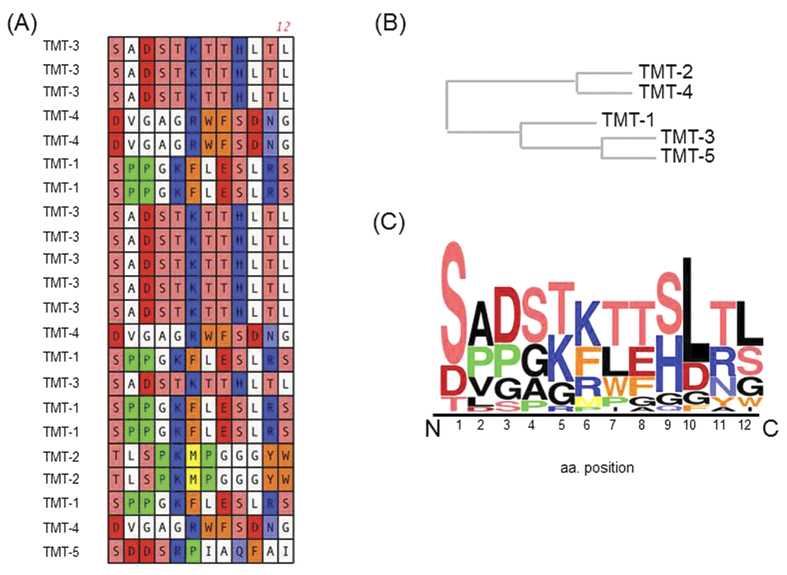
Candidate TM-targeting peptide sequences identified during direct *in vivo* ME phage screening. (**A**) Peptide sequences expressed by bacteriophage recovered from the ME, 1-hr after exposure of the TM. Different colors represent amino acids (aa) with similar characteristics; Blue are basic aa, red are acidic aa, orange are aromatic aa, yellow are sulfur containing aa, polar hydroxylic aa are light red, polar amidic aa are light blue, and green is proline. (**B**) Sequence alignment of the different recovered phage peptides showing a phylogenetic family division. The peptides fall into two phylogenetic families based on amino acid characteristics. (**C**) Frequency of occurrence (Logo plot) of the different amino acids in the phage display selected ligands from Strategy 1.

After selection with three sequential applications to the TM of infected rats and recovery from the ME and amplification, 22 phage clones were selected. The deduced amino acid sequences displayed of these phage clones are listed in Table 1. Because of library collapse due to screening enrichment, this resulted in the identification of five unique clones (TMT 1–5, Table 1, Fig. 1). The phylogenetic tree shows that these peptides fall into two groups based on sequence similarity (Fig. 1B). When the sequences were aligned and examined according to their physical and chemical properties, it was evident that there was a strong selection for preferred groups of amino acids as shown in the sequence Logo plot consensus profile (Fig. 1C). The overall height of the stack indicates the sequence conservation at that position, while the height of symbols within the stack indicates the amino acid relative frequency at that position. There was a strong selection for a central positively charged residue, lysine or arginine (K/R), in all five peptides. Lysine and arginine share similar characteristics and are comparable in size and structure, the only difference being that arginine harbors an extra amine group. TMT-1, TMT-3 and TMT-5 contained conserved polar residues at the N-terminus position. Meanwhile, TMT-2 and TMT-4 contained two aromatic residues: Tyrosine (Y) and tryptophan (W) or phenylalanine (F) and tryptophan (W). Both TMT-1 and TMT-2 had a strong preference for a proline (P) residue at position 3.

#### Phage identification strategy 2: Sequential enrichment of phage for binding to the TM, internalization into the TM and then TM transit

**Figure 2:**
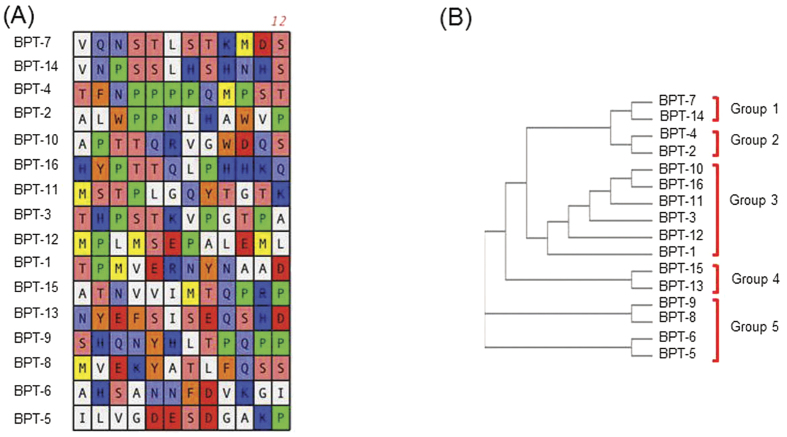
Candidate TM-binding and transiting peptide sequences identified during successive *in vivo* phage screening. (**A**) Peptide sequences expressed by bacteriophage recovered from the ME, 1-hr after exposure of the TM to successively enriched phage display libraries. The color schematics are same as in Fig. 1. (**B**) Phylogenetic tree analysis shows the different family divisions.

After 10 rounds of biopanning (3 rounds of selection for TM binding, 3 rounds of selection for TM internalization and 4 rounds of selection for TM penetration), 30 random clones were sequenced as described earlier. Of these, 16 unique peptides were identified (Table 2, Fig. 2). Analyses of the peptide sequences revealed relative variability between the sequences. Sorting the sequences into groups using ClustalW2 for alignment identified 5 major phylogenetic groups (Fig. 2B). Group 1 contained two peptides that are characterized by a conserved NxSTLSTK core motif. Group 2 had the noteworthy PPxx core motif and a variable C-terminal region. These were different from group 3 which appeared to have a PT(S/T)E(R/K) central motif preceding a variable C-terminal region. The fourth category of sequences contained a polar negatively charged N-terminal region followed by the consensus aliphatic motif SISEQxR though the C-terminal region. The final sequence group contained an interesting HxxYxxD motif and a C-terminal QxP motif. These results indicate that the different grouped peptides could be binding a variety of targets due to the differences in the consensus motifs and the physical and chemical characteristics of the amino acid groups involved. Many of the residues contained are polar, either negatively or positively charged, which suggests that electrostatic interactions are at play.

### Validation of internalization and comparative peptide phage transit rates

**Figure 3:**
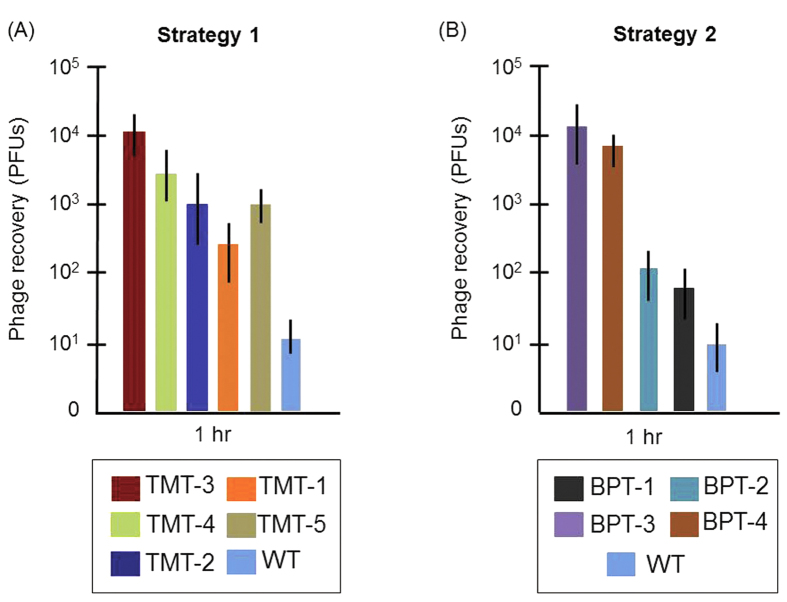
Quantification of amount of translocated phage particles present in the ME, after 1-hr incubation of 10^10^ PFUs of individual phage clones over the TM. The top clones (present at 3 or more copies) identified in the screening from strategies 1 and 2 were compared versus WT phage particles. (**A**) Quantification of transport rate of the five phage peptides recovered in Strategy 1. Phage particles bearing TMT-3 peptide were recovered the greatest compared to WT phage or phage bearing the other peptides. (**B**) Comparison of 1-hr transport of selected phage recovered in Strategy 2 with that of TMT-3 identifies two additional peptides that mediate comparable transport rates. Values represent the mean and SD from six animals per condition.

**Figure 4:**
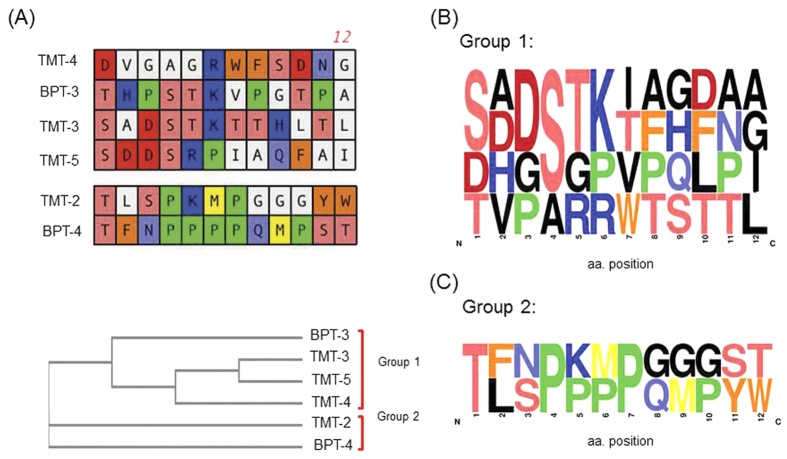
Sequence analysis of TM translocation peptide candidates from the top positive phage clones. (**A**) Phylogenetic tree analysis grouping the different peptide sequences by similarity. Comparison of these six peptides primary sequence reveals the structural similarities in the side chains. The color schematics are same as in Fig. 1. (**B,C**) Consensus Logo plot contour of the two families of peptides that target TM showing the core motifs identified for group 1: “ST(K/R)T” and group 2: “PxxP”.

We quantified the rate of TM transit into the ME for all peptides identified by Strategy 1 (Fig. 3A) and those from Strategy 2 (Fig. 3B) that appeared at a frequency of three or more in our sequenced sample (Tables 1 and 2). Individual phage clones were amplified and 1 × 10^10^ phage particles were applied onto one TM of six anesthetized rats for 1 hr. The number of phage particles recovered from the ME was then compared to that observed for WT (untargeted) phage. All selected phage peptide particles displayed TM-transit rates significantly greater than that of WT phage. The highest rates of transport were observed for phage bearing TMT-2, TMT-3, TMT-4 or TMT-5 peptide from Strategy 1 (Fig. 3A) and phage bearing peptides BPT-3 or BPT-4 from Strategy 2 (Fig. 3B). Interestingly, phylogenetic analysis revealed homology between TMT-3 and BPT-3 (Fig. 4A), even though they were identified using different targeting strategies. On the other hand, the peptide sequence of BPT-4 was clearly distinct in the phylogeny analysis.

Sequence alignment (Fig. 4A) and phylogeny analyses (Fig. 4B) were performed on peptides exhibiting more than 2 × 10^3^ phage particles in the ME after 1 hr incubation of the peptide-phage on the TM. The resulting phylogenetic tree showed that these peptides fell into two groups. Sequences from each group were used to generate sequence Logo plots to identify conserved residues (Fig. 4C). The first set of sequences TMT-3, TMT-4, and BPT-3 displayed the ST(K/R)T core motif noted above. Serines (S) and threonines (T) are quite common in protein binding sites. The hydroxyl group is fairly reactive, being able to form hydrogen bonds with a variety of polar substrates. Lysine and arginine being positively charged also support electrostatic interactions at the binding/active site. The second group (TMT-2 and BPT-4) was markedly different, displaying a PxxPxxP motif. Typically, proline forces a sharp turn or knick in peptide structure, because its unique side chain is bonded to the backbone nitrogen as well as to carbon in a cyclic geometry. Furthermore, PxxP motif typically binds a shallow hydrophobic structural groove with weak affinity, as in the case of many SH3 and WW domains^35^.

### Transport kinetics, saturation and competitive binding analysis

**Figure 5:**
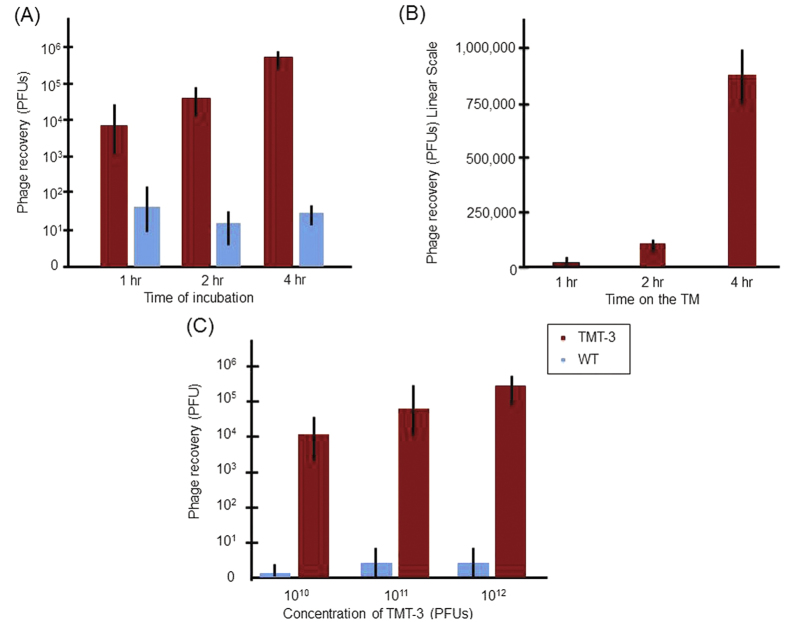
Kinetics of TMT-3 phage translocation and concentration dependence. (**A**) Quantification of TMT-3 phage recovery with respect to increased time. (**B**) A linear X-axis illustrates the superiority of TM-3 phage over WT phage in TM translocation. (**C**) Quantification of TMT-3 phage recovery as increasing amounts of phage were applied to the TM. TMT-3 translocation increases after 1-hr proportionally to concentration and time, indicating that the transport mechanism is not saturated at these copy numbers. Values are the mean and SD number of phage recovered from six animals for each WT and TMT-3 group.

Among the six positive phage clones, TMT-3 had the greatest recovery from the ME (Fig. 3). Therefore, we chose phage bearing TMT-3 and its displayed peptide for further investigation in the following sections. Comparison of phage recovery at 1, 2 and 4 hrs of contact with the infected TM revealed an exponential increase from approximately 10^4^ to nearly 10^6^ phage particles (Fig. 5A). In contrast, recovery of untargeted WT phage was unaffected by time on the TM. When the data are plotted on a linear scale, entry of control WT phage was negligible (Fig. 5B). Studying Fig. 5C, we concluded that the TMT-3 clone phage recovery increased proportionally with concentration, indicating no saturation at the titers tested and where time was limited to 1 hr. This result indicates that the phage peptide particle was utilizing first-order kinetics and that concentration of phage particles inside the ME was directly proportionate to the targeted phage concentration applied on the TM. Applying more of the untargeted WT phage did not increase the random entry of the phage alone, indicating the need to display the TMT-3 peptide for the phage cargo to transit the TM.

**Figure 6:**
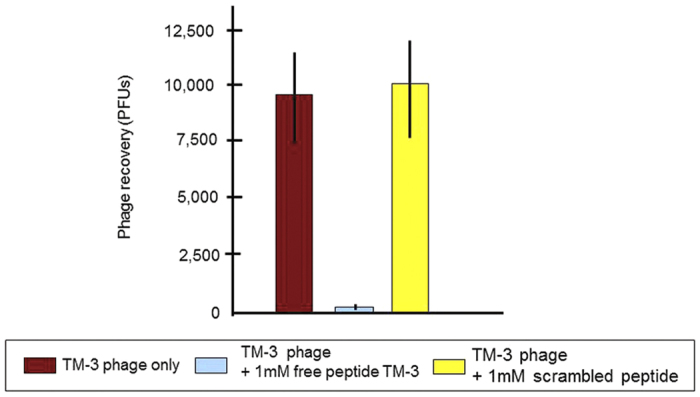
Competitive binding of the synthetic TMT-3 peptide to the TM. The transport of TMT-3 phage was effectively completed by the corresponding synthetic TMT-3 peptide after 1-hr incubation indicating that they could compete for the same binding site. A scrambled synthetic peptide had no effect on the transport of the positive TMT-3 phage clone. Values are the mean and SD number of phages recovered from ME fluids of six rats.

To establish the specificity of translocation, we assessed whether a synthetic TMT-3 peptide would interfere with internalization of corresponding peptide-bearing phage into the ME. Pretreatment of the TM with an excess concentration of synthetic TMT-3 peptide *in vivo* inhibited peptide-associated phage TMT-3 binding and translocation into the ME. A peptide with the same amino acids but scrambled order had no effect (Fig. 6) indicating that TMT-3 phage clone binds to the TM by displaying the TMT-3 peptide and that phage and free peptide compete for the same binding site. In addition, TMT-3 phage was applied onto the TMs of uninfected MEs (n = 6). We observed that for healthy TMs, phage transport into the ME was quite limited when compared to the infected TM (data not shown). This could be attributed to limitations in efficient phage transport when fluid is not present on the other side of the TM. Alternatively, it is possible that the transit mechanism employed is only present in infected TMs.

### Targeted phage utilizes active transport to cross the TM into the ME

**Figure 7:**
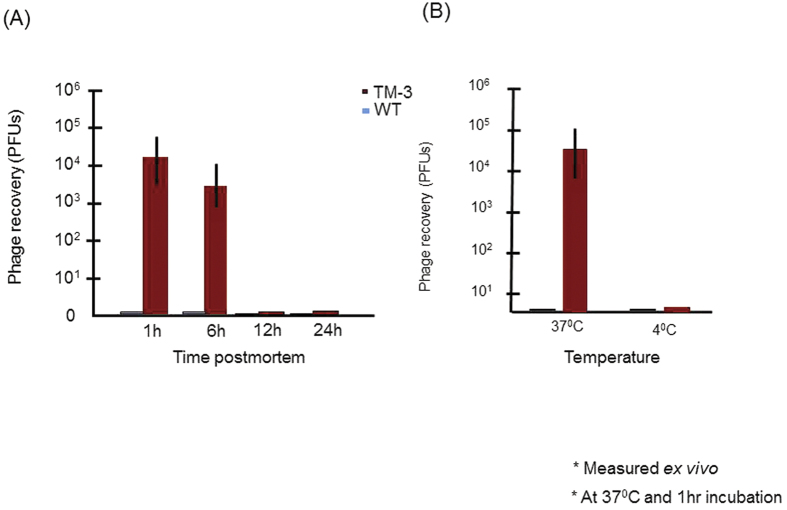
Temperature and metabolic requirement of TM transport. (**A**) TM transport declines after death, suggesting a metabolic requirement. (**B**) Recovery of TMT-3 peptide phage from the ME after incubation of 10^10^ phage particles on the exterior surface of the *ex vivo* for 1-hr at 37 °C versus 4 °C. The temperature dependence of TMT-3 peptide recovery may also suggest active transport across the TM.

In order to learn more about the pharmacokinetics mechanisms of transport, we tested the effect of varying metabolism or temperature on transport rates. Active transport is O_2_ and temperature sensitive. MEs were removed intact from deeply anesthetized rats undergoing OM as described above. Phage bearing TMT-3 were applied for 1 hr on the TMs of the *ex vivo* MEs that had been maintained at 4 °C for either 1, 6, 12 or 24 hrs. At each time point, the TMs were then warmed to 37 °C and incubated for 1 hr with targeted TMT-3 phage. As shown in Fig. 7, while TMs 1 hr after death showed transmigration similar to that observed *in vivo,* by 6 hrs after death the recovery of phage had declined by ~80%. No recovery was observed in TMs stored for 12 or 24 hrs postmortem (Fig. 7A). The fact that *ex vivo* transport occurs for a few hours but then declines strongly suggests active transport. Translocation is also temperature dependent. ME recovery of WT and TMT-3 phage were compared after 1 hr exposure of the TM to 10^10^ PFUs of phage *ex vivo,* at 37 °C or 4 °C. It is well known that endocytosis is inhibited at 4 °C^36^,^37^. As shown in Fig. 7B, the recovery of control WT phage was very low at both temperatures indicating that no microperforations or cross contaminations compromised the experimental design. We noted that at 37 °C, TMT-3 phage recovery was similar to that seen *in vivo*. However, TMT-3 transit was severely compromised at 4 °C, and detected at similar levels to that of the control WT phage (Fig. 7B).

## Discussion

Here we demonstrate the existence of a peptide-dependent mechanism capable of moving particles and/or molecules across an intact un-perforated infected TM and into the ME. The process was uncovered using phage display, an efficient, versatile and rapid means by which to identify compounds with desired biochemical and biological characteristics for an array of targets^24^,^26^,^29^,^38^,^39^. The transport was specific because a synthetic peptide blocked interaction between the phage and the TM. Internalization of cargo was biological because it was inhibited at low temperatures and declined after death. Targeted particle translocation was also time and input number dependent.

In this study, two biopanning protocols were used to identify specific peptides that target transport across the TM. The starting naïve phage library applied to the TM contained 1000 copies of approximately 3 billion unique sequences. In one strategy, we identified peptide targets that were able to cross directly into the ME. This screen identified only four peptide sequences, each present in multiple copies, illustrating the power of this technique to isolate highly specific peptides without prior knowledge of a target transport mechanism. These peptides are extremely rare, in that only 4 out of 2.9 × 10^9^ peptides were obtained. In the alternative biopanning protocol, a successive enrichment strategy was utilized to first select for phage that bind the TM from the original library for three successive rounds. This TM-binding library was then enriched for particles that internalized into the TM epithelium and finally were transported into the ME. More peptides were identified via this screening protocol. Peptide sequences isolated by the two screening methods showed no frank redundancy. However, sequence and phylogeny analyses of the peptides revealed linkages and divided the peptides into four main classes that were represented by a central core group of related amino acid sequence.

The peptides from two screening strategies were analyzed using the SAROTUP suite web tool^34^. This web program determines whether identical or similar peptide sequences have been reported to date by various biopanning experiments deposited by the phage display community, to avoid any possible target-unrelated (i.e., false positive) peptides^40^. All the peptides identified were novel to the database. They also had no known binding targets with the exception of BPT-14, which has been suspected to bind immunoglobulin Fc region motifs^34^. In addition, in checking the MimoDP database, we found that BPT-1, BPT-6 and BPT-12 were previously selected as part of human single-chain variable fragment (HuScFv) which targets Dengue virus envelop protein E in a biopanning study^41^.

The ability to deliver specific targets selectively and locally to the ME has broad implications. These peptides can be used as drug carriers in the development of efficacious target-directed therapies to the ME. Ototopical drug delivery can present a safe and efficient delivery strategy for administration of therapeutics into the ME as an alternative to systemic drugs or surgery. For example, local antibiotic therapy for OM would have the benefit of efficacy while avoiding the side-effects of oral antibiotics and exposure of off-target bacteria and tissues. However, currently available approaches for ME drug delivery rely upon injection through the membrane as the most effective methodology. Other attempts at trans-tympanic drug deliver have employed invasive and noninvasive approaches like passive diffusion aided by hydrogels^42^, nanoparticles^43^, chemical additives^44^ or surfactants^45^, and physical iontophoresis^46^ to increase membrane permeability. These techniques have had limited efficacy clinically due to the low ME concentration of drug molecules achieved, and inability to assist large molecule transit. Additionally, some of these approaches have potentially harmful and deleterious side-effects, require multiple and frequent applications, distort hearing upon application, are cumbersome, and/or limit a patient’s mobility when long-term application is necessary. The development of TM targeting peptides should allow us to overcome many such hurdles by dramatically increasing the efficiency of delivery and providing simplicity of application.

The structure of the trans-tympanic peptides may contain clues as to how transport might occur, although the data are far from conclusive. At a structural level, most of the peptides contain a central lysine residue tightly coordinated by surrounding polar residues (serine or threonine). In a few peptides, an arginine is present at the equivalent position. Both lysine and arginine are positively charged basic amino acids with similar electrostatic interactions and have been previously implicated to be strongly attracted to the negatively charged phosphate groups of the cellular membrane phospholipids^40^. In addition, many ion-gated channels have conserved lysine and arginine residues that play an important role in regulating opening and closing of these channels^47^. The BPT-4 peptide is distinct in structure, in that it contains a stretch of four prolines followed by a fifth proline residue separated by two amino acids. Repetitive proline-rich regions are thought to function as docking sites for several signaling receptor interactions^35^,^48^. In addition, this proline rich motif could form a poly-proline helix^48^. Poly-proline helices are the predominant secondary structure in collagen forming the collagen fibers^49^. Collagen is abundantly present in the TM^50^, making the BPT-4 a very interesting ligand. For example, these poly-proline rich motifs have been shown to interact with Profilin^51^, an actin-binding regulatory protein. Profilins are small proteins that engage in the regulation of actin-based processes, polymerization, membrane trafficking, and nuclear transport^52^. Profilin-2 has been shown to be abundantly present in the human TM in a recent proteomics screen^53^.

During OM, the TM undergoes many structural and biochemical changes^50^. Hence the biological mechanism being employed by some of the peptides identified in our screen could be preferentially present in an infected TM. Our results with healthy TMs suggest this possibility. Currently, our laboratory is performing screens using healthy TMs to explore the possibility of phage transport into the ME. As the healthy (uninfected) ME cavity is filled with air, there might also be a need for fluid to mediate efficient phage transport across the TM. This avenue is also being explored.

The nature and function of the identified active TM transport mechanism involved in phage peptide transport through the TM identified is unclear. The transport mechanism used by the targeted phage is biologically active, in that binding internalization and translocation are dose, temperature and time dependent. Notably, transport is absent more than 6 hrs after death. Finally, competition by synthetic peptides that contain the targeted phage-displayed peptide sequence to compete with phage for binding implicates a receptor-mediated process for binding and translocation. While epithelia form protective barriers throughout the body, trans-epithelial transport of molecules and cells is common^54^, and is often required for normal epithelial function. For example, transport of antibodies and surveillance immune cells is part of the normal biology of all mucosae (e.g., gut and ME). However, the TM is distinct from many such barriers in that it consists of three different layers that need to be crossed by any target: an epidermal outer layer, richly supplied with tight junctions; a connective tissue layer which while dense is extracellular and does not provide a robust diffusion barrier; and the squamous mucosal epithelial layer which also has tight junctions interconnecting the cells, but which is oriented oppositely to the epidermal epithelium. One possibility for active transport is paracellular. The use of paracellular tight-junctions as receptors is a common strategy for small molecules such as glucose, drugs and viruses to facilitate transmission across epithelial barriers^55^. Leukocytes are also able to transit epithelial barriers via paracellular transport^56^, separating intercellular junctions in the process, to cross into mucosal lumens such as in the ME cavity during OM^57^. An alternative mechanism is trans-cellular transport, using cell surface receptors, which can occur in either a basolateral or laterobasal direction^54^. While molecules can move into and through a cell by passive diffusion, they are moved far more efficiently by active transport, including carrier- and receptor- mediated systems. Carrier-mediated systems employ energy-dependent transporter proteins anchored to the cellular membrane to bring substrates across the membrane into the cell. These systems are involved in the active transport of many important nutrients and small molecules such as vitamins, sugars, and amino acids, as well as drugs. On the other hand, receptor-mediated transport systems utilize substrate binding to trigger an encapsulation and cytosis process that results in the formation of transport vesicles that carry the cargo substrate (and sometimes other molecules) into and through the cell, through endocytosis, exocytosis and transcytosis^58^. These types of mechanisms usually enable the transport of larger molecules than carrier-mediated systems. Conversely, while peptide transport across the TM appears to be active, we cannot yet identify whether either of these potential mechanisms, or yet another, is involved. However, receptor identification through biochemical processes is currently underway to identify the specific transport mechanism and the molecular apparatus that operates in trans-TM transport. Nonetheless, the peptides can still be developed for drug targeting. On a final note, it is interesting to highlight that the peptides identified here represent a first-generation of candidate sequences for further optimization and conversion to leads. Additional studies are currently underway to identify the specific transport mechanism and the molecular apparatus that operates in trans-TM transport.

## Additional Information

**How to cite this article**: Kurabi, A. *et al*. Discovery of a Biological Mechanism of Active Transport through the Tympanic Membrane to the Middle Ear. *Sci. Rep.* **6**, 22663; doi: 10.1038/srep22663 (2016).

## Figures and Tables

**Table 1:** Summary of the four unique isolated TM transmigrating peptide sequences isolated using strategy 1 (direct entry to ME), and their characteristics.

Peptide	Frequency	Sequence	Numbers of functional amino acids			pI
			Basic	Acidic	Hydrophobic	
TMT-1	6/22	SPPGKFLESLRS	2	1	4	8.46
TMT-2	2/22	TLSPKMPGGGYW	1	0	4	8.26
TMT-3	10/22	SADSTKTTHLTL	2	1	2	6.64
TMT-4	4/22	DVGAGRWFSDNG	1	2	1	4.21
TMT-5	NA	SDDSRPIAQFAI	1	2	1	3.92

**Table 2:** Summary of the 16 unique isolated TM binding, penetrating and then transiting peptide sequences isolated using strategy 2 (sequential binding, internalization and then entry to ME), and their characteristics.

Peptide	Frequency	Sequence	Numbers of functional amino acids			pI
			Basic	Acidic	Hydrophobic	
BPT-1	5/30	TPMVERNYNAAD	1	2	4	4.37
BPT-2	3/30	ALWPPNLHAWVP	1	0	5	6.79
BPT-3	3/30	THPSTKVPGTPA	2	0	4	8.44
BPT-4	3/30	TFNPPPPQMPST	0	0	6	5.19
BPT-5	2/30	ILVGDESDGAKP	1	3	4	4.03
BPT-6	1/30	AHSANNFDVKGI	2	1	2	6.79
BPT-7	2/30	VQNSTLSTKMDS	1	1	3	5.81
BPT-8	1/30	MVEKYATLFQSS	1	1	3	5.75
BPT-9	1/30	SHQNYHLTPQPP	2	0	4	6.66
BPT-10	1/30	APTTQRVGWDQS	1	1	2	5.88
BPT-11	2/30	MSTPLGQYTGTK	1	0	3	8.34
BPT-12	1/30	MPLMSEPALEML	0	2	6	3.79
BPT-13	1/30	NYEFSISEQSHD	1	3	1	4.13
BPT-14	1/30	VNPSSLHSHNHS	3	0	3	7.00
BPT-15	1/30	ATNVVIMTQPRP	1	0	6	9.79
BPT-16	2/30	HYPTTQLPHHKQ	4	0	3	8.61

## References

[b1] Thomas N. M. & Brook I. Otitis media: an update on current pharmacotherapy and future perspectives. Expert Opin Pharmacother. 15, 1069–1083 (2014). 24793547 10.1517/14656566.2014.903920

[b2] Lieberthal A. S. . The Diagnosis and Management of Acute Otitis Media. Pediatrics. 131, e964–e999 (2013). 23439909 10.1542/peds.2012-3488

[b3] Grijalva C. G., Nuorti J. & Griffin M. R. Antibiotic prescription rates for acute respiratory tract infections in US ambulatory settings. JAMA. 302, 758–766 (2009). 19690308 10.1001/jama.2009.1163 PMC4818952

[b4] Rosenfeld R. M. . Clinical practice guideline: Tympanostomy tubes in children. Otolaryngol-Head Neck Surg. 149, S1–S35 (2013). 23818543 10.1177/0194599813487302

[b5] Acuin J. *Chronic suppurative otitis media: burden of illness and management options Geneva, Switzerland: World Health Organization.* (2004) Available at: http://www.who.int/pbd/publications/ Chronicsuppurativeotitis_media.pdf. (Accessed: 26 July 2015).

[b6] Pichichero M. E. Otitis media. Pediatr Clin North Am. 60, 391–407 (2013). 23481107 10.1016/j.pcl.2012.12.007

[b7] Tahtinen P. A. . A placebo-controlled trial of antimicrobial treatment for acute otitis media. N Engl J Med. 364, 116–126 (2011). 21226577 10.1056/NEJMoa1007174

[b8] Johnson C. E. & Belman S. The role of antibacterial therapy of acute otitis media in promoting drug resistance. Paediatr Drugs. 3, 639–647 (2001). 11688595 10.2165/00128072-200103090-00002

[b9] Pichichero M. E. & Reed M. D. Variations in amoxicillin pharmacokinetic/pharmacodynamic parameters may explain treatment failures in acute otitis media. Paediatr Drugs. 11, 243–249 (2009). 19566108 10.2165/00148581-200911040-00003

[b10] van Zon A., van der Heijden G. J., van Dongen T. M., Burton M. J. & Schilder A. G. Antibiotics for otitis media with effusion in children. Cochrane Database Syst Rev. 9, CD009163 (2012). 22972136 10.1002/14651858.CD009163.pub2

[b11] Casey J. R., Adlowitz D. G. & Pichichero M. E. New patterns in the otopathogens causing acute otitis media six to eight years after introduction of pneumococcal conjugate vaccine. Pediatr Infect Dis J. 29, 304–309 (2010). 19935445 10.1097/INF.0b013e3181c1bc48 PMC3959886

[b12] Vergison A. Microbiology of otitis media: a moving target. Vaccine. 26, G5–G10 (2008). 19094935 10.1016/j.vaccine.2008.11.006 PMC7127463

[b13] de Beer B. A. . Hearing loss in young adults who had ventilation tube insertion in childhood. Ann Otol Rhinol Laryngol. 113, 438–444 (2004). 15224825 10.1177/000348940411300604

[b14] Paradise J. L. . Developmental outcomes after early or delayed insertion of tympanostomy tubes. N Engl J Med. 353, 576–586 (2005). 16093466 10.1056/NEJMoa050406 PMC1201478

[b15] Ruah C., Penha R., Schachern P. & Paparella M. Tympanic membrane and otitis media. Acta Otorhinolaryngol Belg. 49, 173–180 (1995). 7610910

[b16] Volandri G., Di Puccio F., Forte P. & Carmignani C. Biomechanics of the tympanic membrane. J Biomech. 44, 1219–1236 (2011). 21376326 10.1016/j.jbiomech.2010.12.023

[b17] Lim D. J. Structure and function of the tympanic membrane: a review. Acta Otorhinolaryngol Belg. 49, 101–115 (1995). 7610903

[b18] Decraemer W. & Funnel W. Anatomical and mechanical properties of the tympanic membrane. In BArs (Ed) Chronic otitis media. Kugler, Amsterdam, pp 51–84 (2008).

[b19] Stenfors L. E., Bloom G. D. & Hellstrom S. The tympanic membrane. Acta Otolaryngol Suppl. 414, 28–30 (1984). 6598267 10.3109/00016488409122877

[b20] Grote J. J., Bakker D., Hesseling S. C. & Van Blitterswijk C. A. Tympanic Membrane Structure during a Staphylococcus Aureus-induced. Middle Ear Infection: A Study in the Rat Middle Ear. Acta Oto-laryngologica. 107, 225–234 (1989). 2929324 10.3109/00016488909127502

[b21] Yan S. D., Qiu Z. M. & Zhou N. S. Ultrastructure of the secondary tympanic membrane in the human fetus. Acta Anat (Basel). 131, 332–337 (1988). 3376739

[b22] Koulich E., Roland P. S. & Pawlowski K. S. Comparison of systemic and otic administration of ofloxacin. Laryngoscope. 120, 2083–2088 (2010). 20830758 10.1002/lary.21088

[b23] Rickles R. J. . Identification of Src, Fyn, Lyn, PI3K and Abl SH3 domain ligands using phage display libraries. EMBO J. 13, 5598–5604 (1994). 7988556 10.1002/j.1460-2075.1994.tb06897.x PMC395523

[b24] Rothe A., Hosse R. J. & Power B. E. *In vitro* display technologies reveal novel biopharmaceutics. FASEB J. 20, 1599–1610 (2006). 16873883 10.1096/fj.05-5650rev

[b25] Nelson A. L., Dhimolea E. & Reichert J. M. Development trends for human monoclonal antibody therapeutics. Nat Rev Drug Discov. 9, 767–774 (2010). 20811384 10.1038/nrd3229

[b26] Pasqualini R. & Ruoslahti E. Organ targeting *in vivo* using phage display peptide libraries. Nature. 1996; 380, 364–366 (1996). 8598934 10.1038/380364a0

[b27] Teesalu T., Sugahara K. N. & Ruoslahti E. Mapping of vascular ZIP codes by phage display. Methods Enzymol. 503, 35–56 (2012). 22230564 10.1016/B978-0-12-396962-0.00002-1

[b28] Gray B. P. & Brown K. C. Combinatorial Peptide Libraries: Mining for Cell-Binding Peptides. Chem Rev. 114, 1020–1081 (2014). 24299061 10.1021/cr400166n PMC4053476

[b29] Kay B. K., Winter J. & McCafferty J. Phage display of peptides and proteins: a laboratory manual Elsevier Science (1996).

[b30] van Rooy I. . Identification of peptide ligands for targeting to the blood-brain barrier. Pharm Res. 27, 673–682 (2010). 20162339 10.1007/s11095-010-0053-6 PMC2837178

[b31] Wu M., Pasula R., Smith P. A. & Martin W. J. 2^nd^. Mapping alveolar binding sites *in vivo* using phage peptide libraries. Gene Ther. 10, 1429–1436 (2003). 12900757 10.1038/sj.gt.3302009

[b32] Costantini T. W. . Targeting the gut barrier: Identification of a homing peptide sequence for delivery into the injured intestinal epithelial cell. Surgery. 146, 206–212 (2009). 19628075 10.1016/j.surg.2009.05.007 PMC4251594

[b33] Huang J. . MimoDB 2.0: a mimotope database and beyond. Nucleic Acids Res. 40, (Database issue) D271-D277, 2012). 10.1093/nar/gkr922 PMC3245166 22053087

[b34] Huang J., Ru B., Li S., Lin H. & Guo F. B. SAROTUP: scanner and reporter of target-unrelated peptides. J Biomed Biotechnol. 2010, 101932 (2010). 20339521 10.1155/2010/101932 PMC2842971

[b35] Kay B. K., Williamson M. P. & Sudol M. The importance of being proline: the interaction of proline-rich motifs in signaling proteins with their cognate domains. FASEB J. 14, 231–241 (2000). 10657980

[b36] Hong G. . Three-dimensional imaging of single nanotube molecule endocytosis on plasmonic substrates. Nat Commun. 3, 700 (2012). 22426221 10.1038/ncomms1698

[b37] Jiao C.-Y. . Translocation and Endocytosis for Cell-penetrating Peptide Internalization. Journal of Biological Chemistry. 284, 33957–33965 (2009). 19833724 10.1074/jbc.M109.056309 PMC2797166

[b38] Omidfar K. & Daneshpour M. Advances in phage display technology for drug discovery. Expert Opin Drug Discov. 10, 651–69 (2015). 25910798 10.1517/17460441.2015.1037738

[b39] Bratkovič T. Progress in phage display: evolution of the technique and its applications. Cell Mol Life Sci. 67, 749–67 (2010). 20196239 10.1007/s00018-009-0192-2 PMC11115567

[b40] Huang J., Ru B. & Dai P. Bioinformatics resources and tools for phage display. Molecules. 16, 694–709 (2011). 21245805 10.3390/molecules16010694 PMC6259106

[b41] Saokaew N. . Human monoclonal single-chain antibodies specific to dengue virus envelope protein. Lett Appl Microbiol. 58, 270–277 (2014). 24266517 10.1111/lam.12186

[b42] Khoo X. . Formulations for Trans-Tympanic Antibiotic Delivery. Biomaterials. 34, 1281–1288 (2013). 23146430 10.1016/j.biomaterials.2012.10.025 PMC3511665

[b43] Al-Mahallawi A. M., Khowessah O. M. & Shoukri R. A. Nano-transfersomal ciprofloxacin loaded vesicles for non-invasive trans-tympanic ototopical delivery: *in-vitro* optimization, *ex-vivo* permeation studies, and *in-vivo* assessment. Int J Pharm. 472, 304–314 (2014). 24971692 10.1016/j.ijpharm.2014.06.041

[b44] Strutz J., Blessing R. & Zollner C. Effects of topical anesthetics on tympanic membrane structure. Arch Otorhinolaryngol. 244, 381–386 (1988). 3348754 10.1007/BF00497470

[b45] Kristinsson K. G., Magnusdottir A. B., Petersen H. & Hermansson A. Effective treatment of experimental acute otitis media by application of volatile fluids into the ear canal. J Infect Dis. 191, 1876–1880 (2005). 15871121 10.1086/430003

[b46] Christodoulou P. . Transtympanic iontophoresis of gadopentetate dimeglumine: Preliminary results. Otolaryngol Head Neck Surg. 129, 408–413 (2003). 14574297 10.1016/S0194-59980300713-7

[b47] Li L., Vorobyov I. & Allen T. W. The different interactions of lysine and arginine side chains with lipid membranes. J Phys Chem B. 117(40), 11906–11920 (2013). 24007457 10.1021/jp405418y PMC6548679

[b48] Harvey Lodish A. B., S. Lawrence Zipursky, Matsudaira P., Baltimore D. & Darnell J. Molecular properties of voltage-gated ion channels in Molecular Cell Biology 4th edn, Section 21.3, NY: (Freeman W. H. ; 2000).

[b49] Zarrinpar A., Bhattacharyya R. P. & Lim W. A. The structure and function of proline recognition domains. Sci STKE. 2003, RE8 (2003). 12709533 10.1126/stke.2003.179.re8

[b50] Shoulders M. D. & Raines R. T. Collagen structure and stability. Annu Rev Biochem. 78, 929–958 (2009). 19344236 10.1146/annurev.biochem.77.032207.120833 PMC2846778

[b51] Stenfeldt K., Johansson C. & Hellström S. The collagen structure of the tympanic membrane: Collagen types i, ii, and iii in the healthy tympanic membrane, during healing of a perforation, and during infection. Arch Otolaryngol Head Neck Surg. 132, 293–298 (2006). 16549750 10.1001/archotol.132.3.293

[b52] Mahoney N. M., Janmey P. A. & Almo S. C. Structure of the profilin-poly-L-proline complex involved in morphogenesis and cytoskeletal regulation. Nat Struct Mol Biol. 4, 953–960 (1997). 10.1038/nsb1197-953 9360613

[b53] Krishnan K. & Moens P. J. Structure and functions of profilins. Biophys Rev. 1, 71–81 (2009). 10.1007/s12551-009-0010-y PMC5425664 28509986

[b54] Britze A., Birkler R. I. D., Gregersen N., Ovesen T. & Palmfeldt J. Large-Scale Proteomics Differentiates Cholesteatoma from Surrounding Tissues and Identifies Novel Proteins Related to the Pathogenesis. PLoS ONE. 9(8), e104103 (2014). 25093596 10.1371/journal.pone.0104103 PMC4122447

[b55] Reuss L. Epithelial transport. Comprehensive Physiology. 309–388 (2011).

[b56] Rajasekaran S. A., Beyenbach K. W. & Rajasekaran A. K. Interactions of tight junctions with membrane channels and transporters. Biochim Biophys Acta. 1778(3), 757–769 (2008). 18086552 10.1016/j.bbamem.2007.11.007

[b57] Shaykhiev R. & Bals R. Interactions between epithelial cells and leukocytes in immunity and tissue homeostasis. J Leukoc Biol. 82, 1–15 (2007). 17452476 10.1189/jlb.0207096

[b58] Jecker P., Pabst R. & Westermann J. Proliferating macrophages, dendritic cells, natural killer cells, T and B lymphocytes in the middle ear and Eustachian tube mucosa during experimental acute otitis media in the rat. Clin Exp Immunol. 126, 421–425 (2001). 11737056 10.1046/j.1365-2249.2001.01543.x PMC1906226

[b59] Rodriguez-Boulan E., Müsch A. & Le Bivic A. Epithelial trafficking: new routes to familiar places. Curr Opin Cell Biol. 16, 436–432 (2004). 15261677 10.1016/j.ceb.2004.06.013

